# S-Equol Protects Chondrocytes against Sodium Nitroprusside-Caused Matrix Loss and Apoptosis through Activating PI_3_K/Akt Pathway

**DOI:** 10.3390/ijms22137054

**Published:** 2021-06-30

**Authors:** Li-Wen Huang, Tzu-Ching Huang, Yu-Chen Hu, Bau-Shan Hsieh, Hsiao-Ling Cheng, Pu-Rong Chiu, Kee-Lung Chang

**Affiliations:** 1 Department of Medical Laboratory Science and Biotechnology, College of Health Sciences, Kaohsiung Medical University, Kaohsiung 80708, Taiwan; lewehu@cc.kmu.edu.tw; 2 Department of Biochemistry, School of Medicine, College of Medicine, Kaohsiung Medical University, Kaohsiung 80708, Taiwan; huangtavia@gmail.com (T.-C.H.); chingshouhu@gmail.com (Y.-C.H.); cprong@gmail.com (P.-R.C.); 3 Graduate Institute of Medicine, College of Medicine, Kaohsiung Medical University, Kaohsiung 80708, Taiwan; hsiehbs@gmail.com; 4 Department of Pharmacy, Kaohsiung Municipal Min-Sheng Hospital, Kaohsiung 80708, Taiwan; chenghl.tanya@gmail.com

**Keywords:** osteoarthritis (OA), S-equol, chondrocyte, PI_3_K/Akt pathway

## Abstract

Osteoarthritis (OA) is a common chronic disease with increasing prevalence in societies with more aging populations, therefore, it is causing more concern. S-Equol, a kind of isoflavones, was reported to be bioavailable and beneficial to humans in many aspects, such as improving menopausal symptoms, osteoporosis and prevention of cardiovascular disease. This study investigated the effects of S-Equol on OA progress in which rat primary chondrocytes were treated with sodium nitroprusside (SNP) to mimic OA progress with or without the co-addition of S-Equol for the evaluation of S-Equol’s efficacy on OA. Results showed treatment of 0.8 mM SNP caused cell death, and increased oxidative stress (NO and H_2_O_2_), apoptosis, and proteoglycan loss. Furthermore, the expressions of MMPs of MMP-2, MMP-3, MMP-9, and MMP-13 and p53 were increased. The addition of 30 μM S-Equol could lessen those caused by SNP. Moreover, S-Equol activates the PI_3_K/Akt pathway, which is an upstream regulation of p53 and NO production and is associated with apoptosis and matrix degradation. As a pretreatment of phosphoinositide _3_-kinases (PI_3_K) inhibitor, all S-Equol protective functions against SNP decrease or disappear. In conclusion, through PI_3_K/Akt activation, S-Equol can protect chondrocytes against SNP-induced matrix degradation and apoptosis, which are commonly found in OA, suggesting S-Equol is a potential for OA prevention.

## 1. Introduction

Osteoarthritis (OA), is a highly prevalent rheumatic musculoskeletal disorder that affected 303 million people globally in 2017 [1]. Women over the age of 50 or those going through the menopause usually have inflammation, aging, or even dead chondrocytes due to estrogen deficiency resulting in OA and usually causing disability of the aged [2,3,4]. Chondrocytes are the most important cells of joints, even if they only account for about 5% of the total volume of cartilage tissue. They play the role of maintaining collagen and aggrecan synthesis of articular cartilage [5]. When chondrocytes become hypertrophic, they will age and trigger apoptosis and a series of cartilage extracellular matrix degradation and osteoarthritis [6]. Apoptotic chondrocytes are frequently found in cartilage lesions and recognized as a signal of OA [7]. 

To release or attenuate the OA-related symptoms become a big issue, and our recent studies have focused on how to attenuate OA progress by nutrient supplementation or by dietary food and concluded that some supplements and food may reduce OA-related unconformable symptoms [8,9,10]. Accumulating studies have exhibited that oxidative stress increases with estrogen deficiency [11], which is related to the pathophysiology of postmenopausal osteoarthritis [12]. In addition, nitric oxide (NO) and reactive oxygen species (ROS) have long been known to be key factors to mediate chondrocyte apoptosis [13]. Excessive NO and ROS may lead to irreversible mitochondrial membrane damage with low potential (ΔΨM), resulting in DNA damage and ultimately cell death [14]. However, current treatments for OA act only on symptoms, not on prevention or cure. The report showed that prevention or reduction in chondrocytes’ apoptosis would be a valid target for modulating cartilage degeneration [6]. 

The soy isoflavone-derived molecule Equol [7-hydroxy-3-(4′-hydroxyphenyl)-chroman] is a daidzein metabolite produced by intestinal microflora. Synthetic Equol exists as two enantiomers, (S)-(−)-Equol and (R)-(+)-Equol, both of which have selective affinity for estrogen receptors (ER) and modulate androgen action, but only S-Equol occurs naturally [15]. S-Equol is well known to have the strongest estrogen receptor binding capacity and has antioxidative activities among all soy isoflavones, such as Genistein, Daidzein, and Biochanin A. Furthermore, its binding capacity is almost equal to 17β-Estradiol [16,17,18]. Rationally, we propose that S-Equol has the potential to mend the symptoms or diseases caused by the deficiency of estrogen. Up to now, it is still unclear whether S-Equol may decrease OA progress or release its related symptoms. Therefore, this study was designed to test the possibility of S-Equol to serve as a protective agent against OA.

Sodium nitroprusside (SNP) is widely used as a donor for NO production in cell culture including articular chondrocytes [6,19,20]; moreover, SNP-treated chondrocytes are used as an in vitro model of OA [21,22,23]. We used the SNP-treated chondrocytes culture to investigate the effects of S-Equol on OA progress and the underline mechanisms.

## 2. Results

### 2.1. S-Equol Reduces SNP-Induced Cell Death

**Figure 1:**
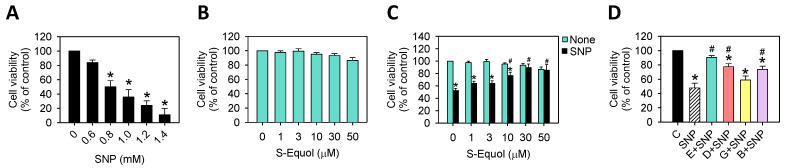
Effects of SNP and/or isoflavones on the viability of primary chondrocytes. (**A**) The cells were treated with 0.6–1.4 mM SNP for 24 h; (**B**) The cells were treated with 1–50 μM S-Equol for 24 h; (**C**) The cells were treated with 1–50 μM S-Equol for 24h in the presence or absence of 0.8 mM SNP for another 24 h; and (**D**) The cells were treated with 30 μM S-Equol (E), Daidzein (D), Genistein (G), or Biochanin A (B) for 24 h in the absence (control) or presence of 0.8 mM SNP for another 24 h. The results are expressed as the mean ± the S.D. for three separate experiments. * *p* < 0.05 compared to the untreated control group. # *p* < 0.05 compared to the SNP treated group.

Firstly, we tested the cytotoxicity of SNP or S-Equol to rat primary chondrocytes. Cells were incubated for 24 h alone or in the presence of 0.6–1.4 mM SNP or 1–50 μM S-Equol and then the cell viability was determined. As Figure 1A shows, SNP induced cell death in a dose-dependent manner at the concentrations of 0.8–1.4 mM, whereas S-Equol showed almost no cytotoxicity (Figure 1B). We chose 0.8 mM SNP to mimic OA conditions for the following experiments because it caused 50% cell viability. As cotreatment with SNP and S-Equol, we observed that S-Equol could reduce SNP-caused cell death and 30 μM S-Equol reached the maximal effect (Figure 1C), thus, the concentration of 30 μM S-Equol was chosen for the following experiments. In addition, we compared the protective effects of S-Equol with other soy isoflavones of Daidzein, Genistein, and Biochanin A on SNP cytotoxicity. Figure 1D showed, at the same concentration of 30 μM isoflavones, S-Equol had the best protective effect among them all.

### 2.2. S-Equol Decreases SNP-Induced Apoptosis

**Figure 2:**
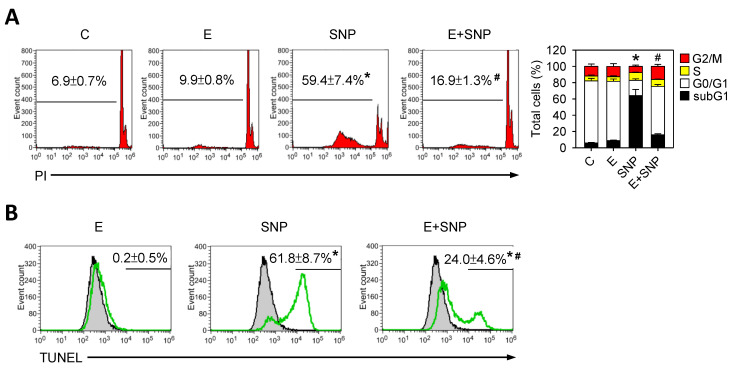
The effect of SNP and/or S-Equol on cell cycle progression and apoptosis of primary chondrocytes. After cells were treated with S-Equol for 24 h in the presence or absence of SNP for another 24 h, (**A**) the cell cycle distribution was determined by flow cytometry and percentage of the subG1 phase was recognized as apoptotic cells; (**B**) apoptotic cells were also determined by TUNEL. The gray peak is vehicle control and the percentage of apoptotic cells of treated cells is shown in the figure. The results are expressed as the mean ± S.D. for three separate experiments, each in triplicate. * *p* < 0.05 compared to the untreated control. # *p* < 0.05 compared to the SNP treated group.

After treatment with 0.8 mM SNP for 24 h in the presence or absence of 30 μM S-Equol, we found that the cell cycle distribution percentage was significantly changed by SNP, whereas the addition of S-Equol could inhibit this change (Figure 2A). Apparently, the subG1 phase distribution, recognized as apoptotic cells, was increased by SNP and that was significantly decreased as S-Equol presented (Figure 2A), indicating that S-Equol could attenuate the SNP-induced apoptosis. Similar results were found in the TUNEL staining apoptotic experiment (Figure 2B).

### 2.3. S-Equol Diminishes the SNP-Caused Decrease of Mitochondrial Membrane Potential (ΔΨM)

**Figure 3:**
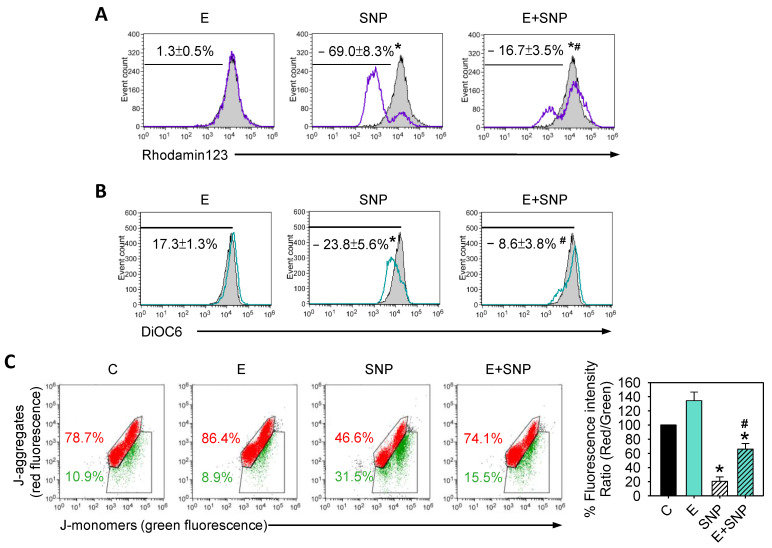
The effect of SNP and/or S-Equol on the mitochondrial membrane potential (ΔΨM) of primary chondrocytes. After cells were treated with S-Equol for 24 h in the presence or absence of SNP for another 24 h, ΔΨM was determined by (**A**) Rhodamin123, (**B**) DiOC6, or (**C**) JC-1 staining, respectively, followed by flow cytometry. The gray filled area is the untreated control, and those delimited by the purple or green lines are the treated groups. In non-damaged cells with high ΔΨM, JC-1 spontaneously forms complexes known as J-aggregates (red fluorescence), whereas in unhealthy cells with low ΔΨM, JC-1 remains in the monomeric form (green fluorescence). The results are expressed as the mean ± S.D. for three separate experiments. * *p* < 0.05 compared to the untreated control. # *p* < 0.05 compared to the corresponding SNP-treated group.

To determine whether SNP and/or S-Equol affected ΔΨM or not, primary chondrocytes were treated with 0.8 mM SNP and/or 30 μM S-Equol for 24 h, and then ΔΨM was measured. These three methods of ΔΨM measurement showed SNP would cause ΔΨM to decrease and S-Equol addition might diminish the decrease (Figure 3A–C). This suggests that S-Equol can lessen SNP-caused mitochondrial membrane damage.

### 2.4. Effect of SNP/S-Equol on Apoptosis-Related Proteins Expressions

Reports showed a reduction in ΔΨM accompanied intrinsic apoptosis [24] in which the functional consequence of pro-apoptotic signaling is mitochondrial membrane perturbation and release of cytochrome *c* to the cytoplasm, where it forms a complex or apoptosome with apoptotic protease activating factor 1 (APAF1) and the inactive form of caspase-9. This complex hydrolyzes adenosine triphosphate to cleave and activate caspase-9. The initiator caspase-9 then cleaves and activates the executioner caspases-3. Caspase-3 promotes the typical apoptosis features, including DNA fragmentation and cell death [25]. Bcl-2 family proteins regulate the release of apoptogenic cytochrome *c* [26]. There is a wealth of experimental evidence that pro-apoptotic BH3-only members of the Bcl-2 protein family (BIM, PUMA, and NOXA) facilitate the apoptotic response by competitively binding to the anti-apoptotic Bcl-2 family members (Bcl-2 and Bcl-X_L_) and displacing them from the pro-apoptotic members, BAX and BAK [27]. BAX and BAK oligomerize to cause mitochondrial outer membrane permeabilization (MOMP), triggering release of cytochrome *c*, and the consequent activation of effector caspases.

**Figure 4:**
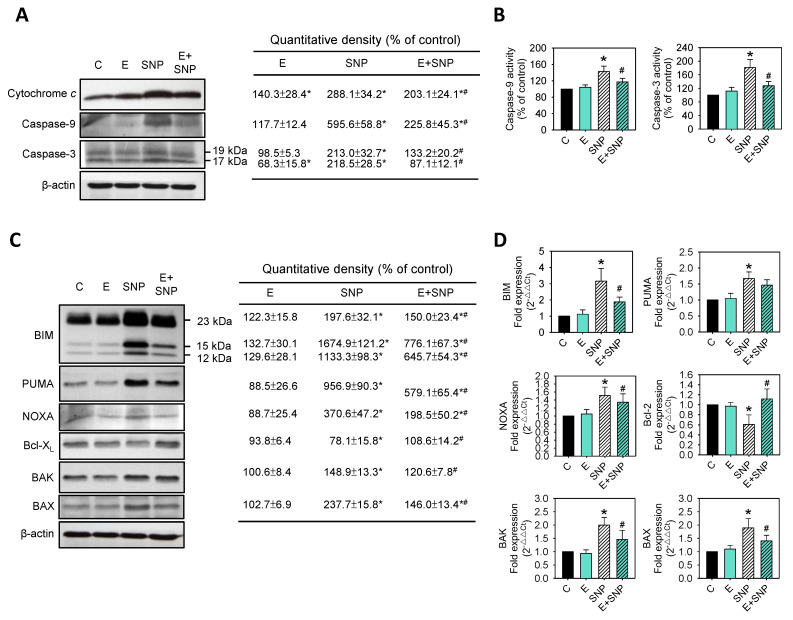
The effect of SNP and/or S-Equol on expressions of apoptotic and anti-apoptotic proteins of primary chondrocytes. After cells were treated with S-Equol for 24 h in the presence or absence of SNP for another 24 h, (**A**) cytochrome *c*, caspase-9, and caspase-3 levels were measured by Western blotting. β-actin was used as the internal control. The data in the right panel are expressed as the relative density compared to the untreated cells (control), which was 100%; (**B**) caspase-9 and caspase-3 activity were measured by a colorimetric assay; (**C**) BIM, PUMA, NOXA, Bcl-X_L_, BAK, and BAX protein levels were measured by Western blotting; and (**D**) BIM, PUMA, NOXA, Bcl-2, BAK, and BAX mRNA levels were measured by real-time PCR. β-actin was used as the internal control. The data are expressed as fold changes compared to that in untreated control, which was 100%. The results are the mean ± S.D. for three separate experiments. * *p* < 0.05 compared to the corresponding untreated control. # *p* < 0.05 compared to the corresponding SNP treated group.

The above pro-apoptotic and anti-apoptotic members were analyzed after SNP and/or S-Equol treatment for 24 h. Clearly, cytochrome *c* levels, and caspase-9 or caspase-3 levels and activities were increased by SNP and these increases were weakened by S-Equol addition (Figure 4A,B). Those were consistent with apoptosis (Figure 2) and mitochondrial membrane potential (Figure 3) results. Furthermore, SNP increased the gene and protein expression of pro-apoptotic BIM, PUMA, NOXA, BAX, BAK, and decreased anti-apoptotic proteins expressions of Bcl-X_L_ and Bcl-2 (Figure 4C,D). Similar to the above findings, S-Equol addition could lessen all the SNP-caused effects.

### 2.5. S-Equol Inhibits SNP-Induced Matrix Degradation

Type II collagen and aggrecan are the main extracellular matrix (ECM) proteins in cartilage [28]. MMP-13 is a product of chondrocytes that reside in cartilage. In addition to degrading collagen, MMP-13 also degrades the proteoglycan molecule aggrecan, thus playing a dual role in matrix destruction. In addition, the expression of other MMPs, such as MMP-2, MMP-3, and MMP-9, is elevated in arthritis, and these enzymes degrade non-collagen matrix components of joints [29].

**Figure 5:**
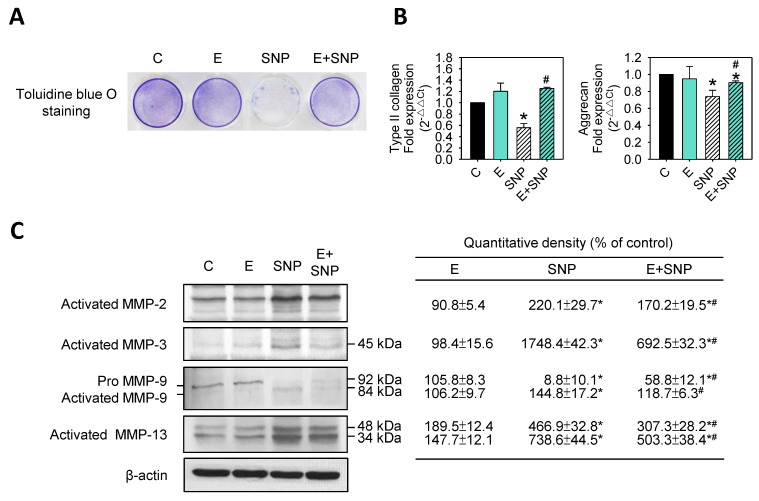
The effect of SNP and/or S-Equol on extracellular matrix and MMPs expression of primary chondrocytes. After cells were treated with S-Equol for 24 h in the presence or absence of SNP for another 24 h, (**A**) glycosaminoglycan (GAG) contents were determined by toluidine blue O staining followed by colorimetric assay; (**B**) mRNA levels of type II collagen and aggrecan were measured by real-time PCR and β-actin was used as the internal control. The data are expressed as fold changes compared to those in untreated control; (**C**) MMP-2, MMP-3, MMP-9, and MMP-13 protein levels were measured by Western blotting and β-actin was used as the internal control. The data of the right panel are expressed as the relative density compared to that in untreated cells (control), which was 100%. The results are the mean ± S.D. for three separate experiments. * *p* < 0.05 compared to the corresponding untreated control. # *p* < 0.05 compared to the corresponding SNP treated group.

To determine whether matrix synthesis and degradation was affected by SNP and/or S-Equol, primary chondrocytes were treated with 0.8 mM SNP and/or 30 μM S-Equol for 24 h, then polysaccharides contents were examined by toluidine blue O staining, type II collagen, and aggrecan gene expression were analyzed by real-time PCR, and MMP-2, MMP-3, MMP-9, and MMP-13 protein expressions were analyzed by Western blotting. Results showed SNP significantly decreased acidic polysaccharides levels (Figure 5A), and gene expressions of type II collagen and aggrecan (Figure 5B), whereas the addition of S-Equol could significantly lessen the decrease of matrix synthesis. Figure 5C showed that SNP could dramatically increase the activation of MMP-2, MMP-3, MMP-9, and MMP-13, and the addition of S-Equol could lessen this trend. This suggests that S-Equol may decrease the SNP-caused matrix loss.

### 2.6. S-Equol Reduces SNP-Induced NO and H_2_O_2_ Production

**Figure 6:**
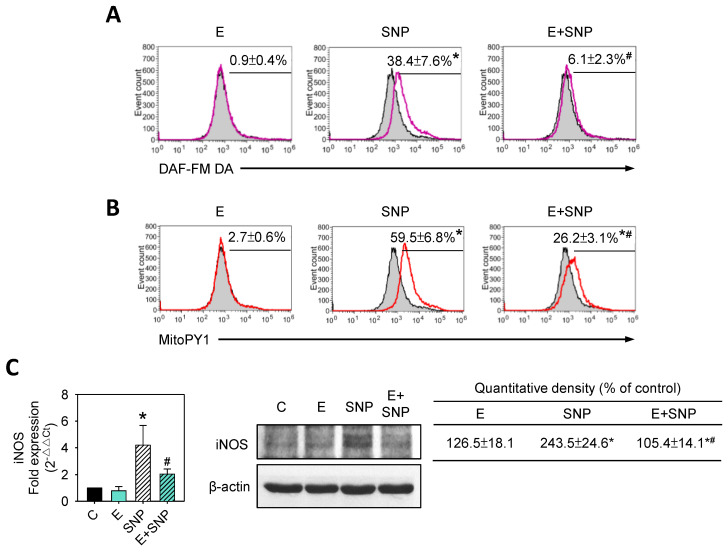
The effect of SNP and/or S-Equol on nitric oxide (NO) and hydrogen peroxide (H_2_O_2_) production of primary chondrocytes. After cells were treated with S-Equol for 24 h in the presence or absence of SNP for another 24 h, (**A**) NO or (**B**) H_2_O_2_ production was determined by DAF-FM DA or MitoPY1 staining, respectively, followed by flow cytometry. The gray filled area is the untreated control, and those delimited by the purple or red lines are the treated groups. (**C**) iNOS mRNA (left side) and protein (right side) levels were measured by real-time PCR and Western blotting. β-actin was used as the internal control. The data are expressed as fold changes compared to that in untreated control. The results are expressed as the mean ± S.D. for three separate experiments. * *p* < 0.05 compared to the untreated control. # *p* < 0.05 compared to the corresponding SNP treated group.

Some studies indicated that the productions of nitric oxide (NO) and hydrogen peroxide (H_2_O_2_) could explain the cytotoxicity of SNP [20,30] and enhancement of inducible nitric oxide synthase (iNOS) related to NO production [31]. To determine whether NO, H_2_O_2_, and iNOS were involved in the effects of SNP on primary chondrocytes, cells were treated with 0.8 mM SNP and/or 30 μM S-Equol for 24 h, and then intracellular NO generation, mitochondrial H_2_O_2_ production and iNOS expression were measured. Figure 6A,B showed that the NO and H_2_O_2_ levels were significantly increased by SNP treatment, whereas both increases were lowered as S-Equol was added. Both mRNA and protein expressions of iNOS were increased by SNP treatment, while the addition of S-Equol might significantly inhibit the increase of iNOS or even the protein levels, which were lower than in the control group (Figure 6C).

### 2.7. The Effect of SNP/S-Equol on PI_3_K-Akt Pathway

It is known that p53 induces apoptosis of cells mainly by direct or indirect transcriptional activation of the pro-apoptotic proteins BIM, PUMA, NOXA, BAK, and BAX [27,32]. The p53 response is through regulation of the PI_3_K-Akt pathway. When PI_3_K is activated, it will activate the phosphorylation of serine 473 (Ser473) of Akt, which can regulate the phosphorylation of serine 166 (Ser166) of Mdm2 [33]. The phosphorylated Mdm2 can enter the nucleus and bind with p53 to inhibit its transcriptional activity, or the activity of E3 ubiquitin ligase sends p53 to the protesome for ubiquitin-dependent degradation, thereby reducing the expression of p53 in cells to avoid the occurrence of p53-dependent cell apoptosis and maintaining cell survival [34]. Accordingly, we analyzed the PI_3_K-Akt pathway-related parameters.

**Figure 7:**
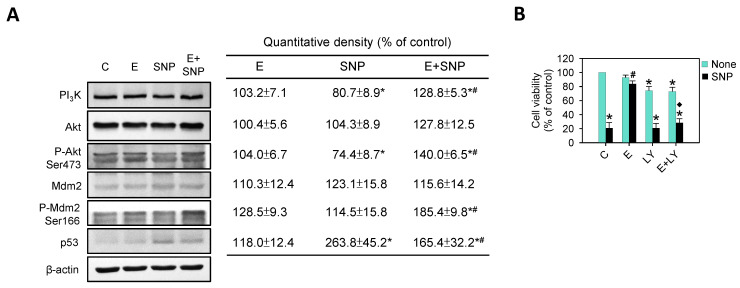
The effects of SNP and/or S-Equol on PI_3_K-Akt pathway of primary chondrocytes. The cells were treated with S-Equol for 24 h in the presence or absence of SNP for another 24 h, and then (**A**) PI_3_K, Akt, P-Akt, Mdm2, P-Mdm2, and p53 protein levels were measured by Western blotting and β-actin was used as the internal control. The data in the right panel are expressed as the relative density compared to the untreated cells (control), which was 100%. (**B**) The cells were pretreated with the 10 μM PI_3_K inhibitor, LY294002 (LY), for 1 h and then treated with 0.8 mM SNP and/or 30 μM S-Equol for a further 24 h and, subsequently, cell viability was measured using trypan blue exclusion. The data are expressed as a percentage of the control group. The results are the mean ± S.D. for three separate experiments. * *p* < 0.05 compared to the corresponding untreated control. # *p* < 0.05 compared to the corresponding SNP treated group. ^◆^ *p* < 0.05 compared to the corresponding S-Equol combined with SNP treated group.

Figure 7A shows that SNP significantly increased p53 expression, while S-Equol addition could lessen this increase, but was still higher than the control group. In contrast, SNP significantly decreased PI_3_K and P-Akt. Simultaneously, S-Equol addition could increase not only PI_3_K and P-Akt but also P-Mdm2 levels. That indicates the PI_3_K-Akt pathway could be activated by S-Equol addition. In order to further clarify the role of PI_3_K-Akt activation by S-Equol addition, primary chondrocytes were pre-treated with a PI_3_K inhibitor, LY294002, for 1 h and then treated with 0.8 mM SNP and/or 30 μM S-Equol for 24 h, then viable cells were analyzed. As Figure 7B shows, the cell viability was different in SNP with or without S-Equol addition, but this difference disappeared as pre-treatment with PI_3_K inhibitor. This suggests that the protective effect of S-Equol addition against SNP-caused cell death is related to PI_3_K -Akt activation.

## 3. Discussion

**Figure 8:**
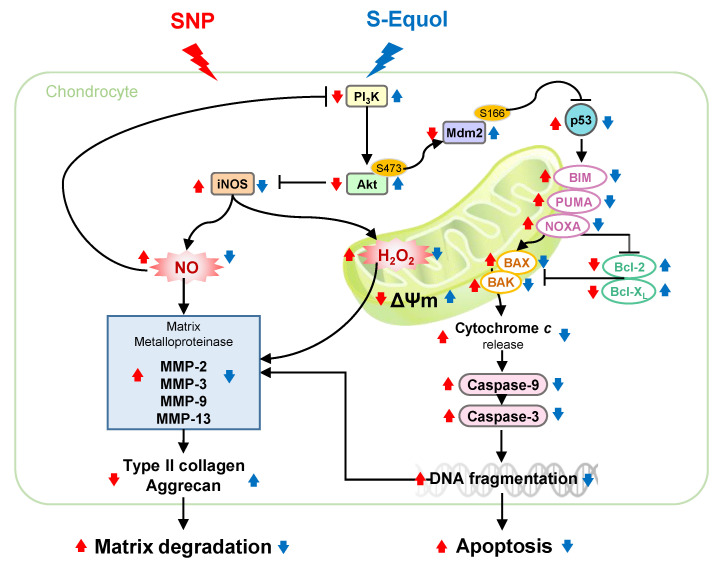
Schematic of S-Equol protects chondrocyte against SNP’s damage. Treatment of chondrocyte with SNP could increase p53, intracellular NO and H_2_O_2_ levels and decrease mitochondrial membrane potential (ΔΨM). Furthermore, the NO could activate MMPs, resulting in type II collagen and aggrecan degradation. Simultaneously, p53 could increase expressions of pro-apopototic proteins including BIM, PUMA, NOXA, BAX, and BAK, and cause a decrease in anti-apoptotic proteins including Bcl-X_L_ and Bcl-2 expression, resulting in the release of cytochrome *c*, activation of caspase-9 and caspase-3, and finally the induction of apoptosis. The addition of S-Equol could lessen those effects caused by SNP. Moreover, S-Equol could active the PI_3_K/Akt pathway, an upstream of apoptosis and NO production, including an increase in phosphorylation of Akt and Mdm2 to inhibit p53 and NO production, by which the apoptosis and matrix degradation caused by SNP are decreased. Red 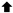: enhanced by SNP; Red 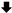: decreased by SNP; Blue 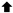: enhanced by S-Equol; Blue 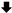: decreased by S-Equol.

The present study showed that, in rat primary chondrocyte, treatment of 0.8 mM SNP causes cell death and an increase in oxidative stress (NO and H_2_O_2_), apoptosis, and proteoglycan loss. Furthermore, the expressions of MMPs of MMP-2, MMP-3, MMP-9, and MMP-13 and p53 are increased. The addition of 30 μM S-Equol can lessen those caused by SNP. Moreover, we found that S-Equol can activate phosphorylation of Akt and Mdm2; in other words, S-Equol activates the PI_3_K/Akt pathway which is an upstream regulation of p53 and NO production, and associated with the occurring of apoptosis and matrix degradation (Figure 8). As pre-treatment of phosphoinositide 3-kinases (PI_3_K) inhibitor, all the S-Equol protection against SNP reduced or disappeared. These results suggest that S-Equol could protect chondrocytes from SNP-induced matrix degradation and apoptosis through activation of PI_3_K/Akt pathway.

It has been reported that SNP can be used as an exogenous NO donor to evoke intracellular apoptosis and MMPs expressions [35]. Owing to the fact that these characteristics are similar to the conditions of OA progress, it has been widely used as an agent to treat chondrocytes to mimic the progress of OA [36]. Consistent with the report, this study showed, after treatment of SNP, chondrocytes not only had higher NO levels, but also high H_2_O_2_, levels resulting in high oxidative stress. Additionally, when more MMPs expressions were found, the apparent circumstances of matrix degradation and apoptosis of chondrocyte found in OA occurred. Although this study could not clarify whether the matrix degradation and apoptosis are directly or indirectly related to NO and/or H_2_O_2_, there was a report that indicated that NO was not only involved in SNP-induced chondrocyte apoptosis but also related to MMPs activities [20]. However, it is well known that increased intracellular oxidative stress leads to apoptosis induction [37]. Herein, we found the decreased mitochondrial membrane potential (ΔΨM) and anti-apoptotic proteins expression and the increased pro-apoptotic proteins, caspase, and cytochrome *c* release after SNP treatment.

The soy isoflavone-derived molecule Equol [7-hydroxy-3-(4′-hydroxyphenyl)-chroman], a daidzein metabolite produced by intestinal microflora, is well known to have the strongest estrogen receptor binding capacity and antioxidative activities among all soy isoflavones; moreover, its binding capacity is almost equal to 17β-Estradiol [16,17,18]. It is already known 17β-Estradiol can promote cell survival and antioxidant activity of chondrocytes via the phosphatidylinositol _3_-kinase-Akt (PI_3_K/Akt) pathway [38,39]. The report indicates that S-Equol can activate the Nrf2/ARE signaling pathway mediated by PI_3_K/Akt to against oxidative stress of endothelial cells [40]. When phosphorylation of Akt at Ser473 becomes active and enhances cell survival, phosphorylation occurs at an Akt consensus site (Ser166) within the Mdm2 protein, a key regulator of p53 function, it subsequently interacts with the tumor suppressor protein p53 to negatively regulate its function. This occurs through two main mechanisms. First, the direct binding of Mdm2 to the N-terminal end of p53 inhibits the transcriptional activation function of p53. Second, Mdm2 possesses E3 ubiquitin ligase activity that targets p53 for modification and subsequent degradation through the 26S proteasome [41,42]. It is known that p53 induces apoptosis of normal cells mainly by directly upregulating the pro-apoptotic genes NOXA, PUMA, and indirectly BIM, and it has been shown to inhibit the expression of Bcl-2 directly [32]. p53 has been reported to migrate to the mitochondria and interact with members of the Bcl-2 family by displacing anti-apoptotic members from pro-apoptotic Bcl-2 proteins, or by directly activating BAX or BAK to induce mitochondrial outer membrane permeabilization (MOMP) [32].

Our results show that addition of S-Equol to SNP-treated primary chondrocytes can lessen all lethal effects caused by SNP and activate PI_3_K/Akt pathway accompanied by increased phosphorylation at Ser473 of Akt and Ser166 of Mdm2, and decreased p53, which is the same as that reported of 17β-Estradiol [38,39]. Accordingly, we propose that S-Equol is a good alternative to 17β-Estradiol. In the experiment of pre-treatment of LY294002, a strong inhibitor of phosphoinositide _3_-kinases, we found the protective effect of S-Equol against SNP disappeared or decreased. This suggests that S-Equol protects chondrocytes from SNP-caused damage through PI_3_K/Akt pathway.

In summary, the present study demonstrates that S-Equol can effectively protect chondrocytes against SNP-induced matrix degradation and apoptosis, which are the common findings of OA progress. That suggests that S-Equol has the potential to be an agent for OA treatment.

## 4. Materials and Methods

### 4.1. Reagents and Antibodies

SNP and all other chemicals of analytical grade were purchased from Sigma-Aldrich Co., LLC. (St. Louis, MO, USA). S-Equol was purchased from Cayman Chemical Company (Ann Arbor, MI, USA). Protein assay reagents were obtained from Bio-Rad Laboratories (Hercules, CA, USA). The following primary antibodies were used in the Western blot analysis. MMP-2, MMP-9, iNOS (NOS2), cytochrome *c*, caspase-9, caspase-3, PUMA, Bcl-X_L_, BAX, p53, MMP-3, and β-actin were purchased from Santa Cruz Biotechnology, Inc. (Dallas, TX, USA). MMP-13, NOXA, and Mdm2 were purchased from Novus Biologicals (Littleton, CO, USA). P-Mdm2 Ser166 was purchased from Abcam plc. (Abcam, Cambridge, UK). BIM, BAK, PI_3_K, Akt, and P-Akt Ser473 were purchased from Cell Signaling Technology (Danvers, MA, USA). Horseradish peroxidase-conjugated anti-mouse, goat, or rabbit IgG antibodies were purchased from Santa Cruz Biotechnology, Inc. (Dallas, TX, USA).

### 4.2. Isolation and Expansion of Rat Primary Chondrocytes

Male and female Sprague Dawley (SD) rats at 8 weeks of age were purchased from BioLASCO Taiwan Co., Ltd. (Charles River Technology, Taipei, Taiwan). Male SD rats at 9 weeks of age (301–325 g) and female SD rats at 9 weeks of age (201–225 g) were used. The present study was performed following the Guide for the Care and Use of Laboratory Animals of the United States National Institutes of Health. The protocol for animal use was reviewed and approved by the Institutional Animal Care and Use Committee (IACUC) of Kaohsiung Medical University (Approval No. 107214; Approval date: 7 May 2019). The extraction of chondrocytes from cartilage was performed as reported previously [43]. Briefly, one litter of 1–2-day-old newborn rats under general anesthesia were used. After disinfecting all animals with 70% ethanol, the skin and soft tissues were removed from the hind legs using sterile scissors and pincer. Then, the femurs were dislocated and the soft tissues discarded. Cartilage sections of femoral heads, femoral condyles, and tibial plateau were immediately harvested using a scalpel and incubated overnight in a thermal incubator under 5% CO_2_, in a petri dish with collagenase D (Roche Diagnostics GmbH, Mannheim, Germany) solution at 0.5 mg/mL in Dulbecco’s modified Eagle’s medium (DMEM). After dispersing the cell aggregates, a suspension of isolated cells yields was carried out. The cell suspension was filtered through a sterile 48 μM cell strainer to obtain chondrocytes. Chondrocytes were seeded on a culture dish in an incubator at 37 °C with 5% CO_2_, and the culture medium was changed every two days. After the cells reached 80–90% confluence, the cultures were trypsinized and the cells were frozen at −80 °C overnight. The cells were stored in liquid nitrogen for long periods. Passage 2 (P2) chondrocytes were analyzed for each experiment.

### 4.3. Cell Culture

The rat primary chondrocytes were cultured at 37 °C with low glucose Dulbecco’s modified Eagle medium (DMEM) containing 100 units/mL of penicillin, 100 μg/mL streptomycin (Gibco BRL, Grand Island, NY, USA), and 10% fetal bovine serum (FBS) (Gibco BRL, Grand Island, NY, USA) in a 5% CO_2_ incubator. The cells were seeded at 5 × 10^5^ onto dishes in DMEM for 16 h to enable attachment and subsequently treated with S-Equol in the presence or absence of SNP for a further indicated period, followed by the analysis of the influences.

### 4.4. Cell Viability Assay

The primary rat chondrocytes (5 × 10^4^/well) were seeded in 24-well plates and incubated with 0–1.4 mM SNP or 0–50 μM S-Equol for 24 h. The cells were harvested, and the viable cells were counted using a dye exclusion technique with 0.4% trypan blue (Gibco BRL, Grand Island, NY, USA) in a hemocytometer. All counts were performed in triplicate.

### 4.5. Effects PI_3_K Inhibitors on Cell Viability

The primary rat chondrocytes (5 × 10^4^/well) were seeded in 24-well plates and pretreated with 10 μM LY294002 (LY) (BioVision, Inc., Mountain View, CA, USA), a PI_3_K inhibitor, for 1 h. Afterward, cells were treated with 30 μM S-Equol for 24 h. Then cells were treated with 0.8 mM SNP for another 24 h, the cell viability was assayed. All counts were performed in triplicate.

### 4.6. Toluidine Blue O Staining

The primary rat chondrocytes (5 × 10^4^/well) were seeded in 24-well plates and treated with S-Equol for 24 h in the presence or absence of SNP for another 24 h. Then, the medium was removed from each well and the cells were washed with PBS. The cells were fixed with 4% paraformaldehyde (PFA) in PBS for 10 min at room temperature and then rinsed with PBS. The fixed cells were incubated with 0.5% toluidine blue O staining solution for 30 min at room temperature. After PBS washes, the image was captured using a digital camera.

### 4.7. Measurement of NO and H_2_O_2_ Levels

The primary rat chondrocytes (5 × 10^5^) were seeded in 6 cm culture dishes and treated with S-Equol for 24 h in the presence or absence of SNP for another 24 h, and then intracellular nitric oxide (NO) and mitochondrial hydrogen peroxide (H_2_O_2_) levels were detected to represent oxidative stress by using fluorescent probes of 4-amino-5-methylamino-2′,7′-difluorofluorescein diacetate (DAF-FM DA) (Cayman Chemical Company, Ann Arbor, MI, USA) [44] and mitochondria peroxy yellow 1 (MitoPY1) (Tocris Bioscience, Bristol, UK) [45], respectively. The detection was performed as described in the manufacturer’s instruction using an Attune NxT, Acoustic Focusing Cytometer (Model AFC2, Thermo Fisher Scientific, Waltham, MA, USA). The data were analyzed using FCSalyzer version 0.9.22 (Sven Mostböck, Vienna, Austria). Each experiment was carried out in triplicate.

### 4.8. Western Blot Analysis

The primary rat chondrocytes (5 × 10^5^) were seeded in 6 cm culture dishes and treated with S-Equol for 24 h in the presence or absence of SNP for another 24 h, and then the cell extracts preparation for Western blot analyses were described previously [45]. Briefly, proteins were separated by 10% SDS-PAGE and then transferred to nitrocellulose membrane (Amersham, GE Healthcare life sciences/Cytiva, Marlborough, MA, USA). After blocking in 2% nonfat milk or 2% bovine serum albumin (BSA) (Sigma-Aldrich Co., LLC., St. Louis, MO, USA), the membranes were incubated with primary antibodies as indicated. The proteins were visualized using chemiluminescence detection (PerkinElmer, Inc., Waltham, MA, USA). β-actin was used as the internal control, and each targeted band was calibrated by the respective β-actin. Afterwards, the data of the study group were quantitatively analyzed relative to the control group. Each experiment was carried out in triplicate.

### 4.9. Quantitative Real-Time PCR Analysis (qPCR)

The primary rat chondrocytes (5 × 10^5^) were seeded in 6 cm culture dishes and treated with S-Equol for 24 h in the presence or absence of SNP for another 24 h, and then the total RNA was isolated by Direct-zol RNA Miniprep Kits (Zymo Research, Irvine, CA, USA) according to the manufacturer’s instructions. Each experiment was carried out in triplicate. The complementary DNA (cDNA) was synthesized from random primed reverse transcription from 2 μg of total RNA using M-MLV reverse transcriptase (Promega Corporation, Madison, WI, USA) according to the manufacturer’s instructions. Real-time PCR was performed on a StepOnePlus Real-Time PCR System (Applied Biosystems, Thermo Fisher Scientific, Waltham, MA, USA) using Fast SYBR Green Master Mix (Applied Biosystems, Thermo Fisher Scientific, Waltham, MA, USA). The mRNAs encoding type II collagen, aggrecan, iNOS, BIM, PUMA, NOXA, Bcl-2, BAK, and BAX were measured using real-time PCR, with β-actin mRNA as the housekeeping gene. The primers and amplified products of each gene used in the present study are shown in Table 1. The cycle threshold (Ct) value of the target gene was normalized to β-actin. The data were calculated and expressed as 2^−ΔΔCt^ [46] using StepOne software version 2.3 (Thermo Fisher Scientific, Waltham, MA, USA).

### 4.10. TUNEL Assay

Apoptosis was determined by the terminal deoxynucleotide transferase-mediated dUTP nick-end labeling (TUNEL) assay. TUNEL was performed using an APO-BrdU™ TUNEL Assay Kit (Thermo Fisher Scientific, Waltham, MA, USA) according to the manufacturer’s protocol and followed by flow cytometric analysis using an Attune NxT, Acoustic Focusing Cytometer (Model AFC2, Thermo Fisher Scientific, Waltham, MA, USA) to quantify apoptosis. The data were analyzed using FCSalyzer version 0.9.22. A minimum of 1 × 10^4^ cells per sample was evaluated in each case [47].

### 4.11. Cell Cycle Progression Analysis

The percentages of cells in the different stages of the cell cycle were estimated by flow cytometric DNA analysis as described previously [48]. A minimum of 1 × 10^4^ cells per sample were evaluated on an Attune NxT, Acoustic Focusing Cytometer (Model AFC2, Thermo Fisher Scientific, Waltham, MA, USA), and the percentages of cells in each cell cycle phase (subG1, G0/G1, S, or G2/M) were determined using FCSalyzer version 0.9.22 (Sven Mostböck, Vienna, Austria). Each experiment carried out in triplicate.

### 4.12. Detection of Mitochondrial Membrane Potential (ΔΨM)

Fluorescent dyes, rhodamin123, 3, 3’-dihexyloxacarbocyanine iodide (DiOC6), and 5, 5’, 6, 6’-tetrachloro-1, 1’, 3, 3’-tetraethylbenzimidazolylcarbocyanine iodide (JC-1), were applied to detect the ΔΨM. The staining procedure was performed according to the manufacture’s protocol. The primary rat chondrocytes (5 × 10^5^) were seeded in 6 cm culture dishes and treated with S-Equol for 24 h in the presence or absence of SNP for another 24 h, and then cells were subsequently stained with 10 μg/mL rhodamin123 (Sigma-Aldrich Co., LLC., St. Louis, MO, USA) for 15 min, 20 nM DiOC6 (Sigma-Aldrich Co., LLC., St. Louis, MO, USA) for 10 min, or 2 μM JC-1 (Thermo Fisher Scientific, Waltham, MA, USA) for 20 min, at 37 °C. A minimum of 1 × 10^4^ cells per sample was then collected, washed in PBS, and evaluated on an Attune NxT, Acoustic Focusing Cytometer (Model AFC2, Thermo Fisher Scientific, Waltham, MA, USA), and analyzed using FCSalyzer version 0.9.22 (Sven Mostböck, Vienna, Austria). Low levels of rhodamin123 or DiOC6 uptake corresponded to a loss of ΔΨM. JC-1 aggregates (red fluorescence) favor high ΔΨM intact cells and, in response to the loss of ΔΨM, JC-1 monomers are formed showing green fluorescence. Cells with collapsed ΔΨM exhibited a decrease in the percentage of red fluorescence and the fluorescence intensity of the red/green ratio. Each experiment was carried out in triplicate.

### 4.13. Measurement of Caspase-9 and Caspase-3 Activities

The primary rat chondrocytes (5 × 10^5^) were seeded in 6 cm culture dishes and treated with S-Equol for 24 h in the presence or absence of SNP for another 24 h, and then the cell pellet was resuspended in lysis buffer and sonicated at 20 s intervals for 2 min on ice. Next, the cell lysate was centrifuged at 12,000× *g* for 10 min at 4 °C, and the supernatant was collected and analyzed immediately for caspase-9 and caspase-3 activity using Caspase-9 Colorimetric Assay Kit (BioVision, Inc., Mountain View, CA, USA) and Caspase-3/CPP32 Colorimetric Protease Assay Kit (Invitrogen, Grand Island, NY, USA), respectively, according to the manufacturer’s instructions.

### 4.14. Statistical Analysis

All data are presented as the means ± standard deviation (S.D.). The differences between control and treated groups were analyzed using ANOVA, followed by Fisher’s Exact Test. All statistical analyses were performed using SAS version 6.011 software (SAS Institute, Inc., Cary, NC, USA). A *p*-value < 0.05 was considered statistically significant. 

## Figures and Tables

**Table 1:** Primer sets for qPCR analysis.

Primer Name	NCBI Reference Sequence	Primer Sequence (5’->3’)
β-actin	EF156276.1	F: ACTATCGGCAATGAGCGGTTCC R: AGCACTGTGTTGGCATAGAGGTC
Type II collagen	NM_012929.1	F: CGAACCCAAAGGACCCAAAT R: TCCGGACTGTGAGGTTAGG
Aggrecan	NM_022190.1	F: CGAGTGAACAGCATCTACC R: GAGTCATTGGAGCGAAGG
iNOS (NOS2)	NM_012611.3	F: GCTACACTTCCAACGCAACA R: ATGGTGAACACGTTCTTGGC
BIM	NM_171988.2	F: GCAAACGATTACCGAGAGGC R: TCCAGACCAGACGGAAGATG
PUMA	NM_173837.2	F: AAGAGCAACATCGACACCGA R: TCCAGGATCCCTGGGTAAGG
NOXA	NM_001008385.1	F: GAGTGCACCGGACATAACTG R: GCTTGGGCTTCTTCTCATCG
Bcl-2	NM_016993.1	F: CTGGGGATGACTTCTCTCGT R: GGACATCTCTGCAAAGTCGC
BAK	NM_053812.1	F: CACCCATGCTCCTGTTATGC R: CCCACCCTTGCTACATTTGG
BAX	NM_017059.2	F: TTGGCGATGAACTGGACAAC R: GTAGAAAAGGGCAACCACCC

iNOS (NOS2): inducible nitric oxide synthase 2; BIM: Bcl-2-like protein 11; PUMA: p53 upregulated modulator of apoptosis; NOXA: phorbol-12-myristate-13-acetate-induced protein 1; Bcl-2: B cell lymphoma 2, apoptosis regulator); BAK: Bcl-2 antagonist/killer 1; BAX: Bcl-2 associated X, apoptosis regulator; F: Forward primer; R: Reverse primer.
